# Mapping complications in thyroid surgery: statistical data are useful for medico-legal management of a recurrent safety issue

**DOI:** 10.1007/s13304-022-01357-8

**Published:** 2022-08-27

**Authors:** Martina Padovano, Matteo Scopetti, Raoul Tomassi, Federico Manetti, Stefano D’Errico, Alessandro Santurro, Giorgio De Toma, Paola Frati, Paolo Miccoli, Vittorio Fineschi

**Affiliations:** 1 Department of Anatomical, Histological, Forensic and Orthopaedic Sciences, Sapienza University of Rome, 00185 Rome, Italy; 2 Department of Medicine, Surgery and Health, University of Trieste, 34149 Trieste, Italy; 3 Department of Medicine, Surgery and Dentistry, University of Salerno, 84081 Salerno, Italy; 4 Department of Surgery, Sapienza University of Rome, 00161 Rome, Italy; 5 Department of Surgical, Medical, Molecular Pathology and Critical Area, University of Pisa, 56126 Pisa, Italy; 6 Department of Medical, Surgical Sciences and Translational Medicine, Sapienza University of Rome, 00189, Rome, Italy

**Keywords:** Thyroid surgery, Litigation management, Thyroid claims, Risk mapping, Quality of health care, Patient safety

## Abstract

**Abstract:**

Quality of care assessment is a crucial tool for patient safety implementation. Litigation relating to thyroid surgery is one of the most represented sectors also due to the continuous increase in the number of thyroid interventions. Given the incidence of the problem, the present study aims to outline an operational methodology for risk mapping and litigation management in thyroid surgery. The study was conducted through the analysis of data collected at Umberto I General Hospital in Rome from 2007 to 2018. All thyroid surgery claims were included and, subsequently, a descriptive statistical analysis of the categorical variables was performed with the representation of frequencies in absolute terms and as a percentage. The results obtained show that in 94% of cases (44 cases) the reported event consists of incorrect treatment. The most frequent adverse events were identified in unilateral or bilateral recurrent nerve lesions (31; 70%); incomplete removal of the thyroid gland (6; 14%), post-surgical hypoparathyroidism (4; 9%), aesthetic damage secondary to surgical scars (2; 5%), dental avulsion during intubation maneuvers (1; 2%). The experience derived from the risk mapping through management of thyroid claims proved it to be a reactive tool of considerable importance in clinical governance. The promotion of measures aimed at improving the satisfaction of some critical parameters identified in the litigation management activity such as adherence to the indications for surgery, the preoperative diagnostic path, and the adequacy of the surgical report allows to further implement the quality of care in the surgical treatment of thyroid pathology.

**Graphical Abstract:**

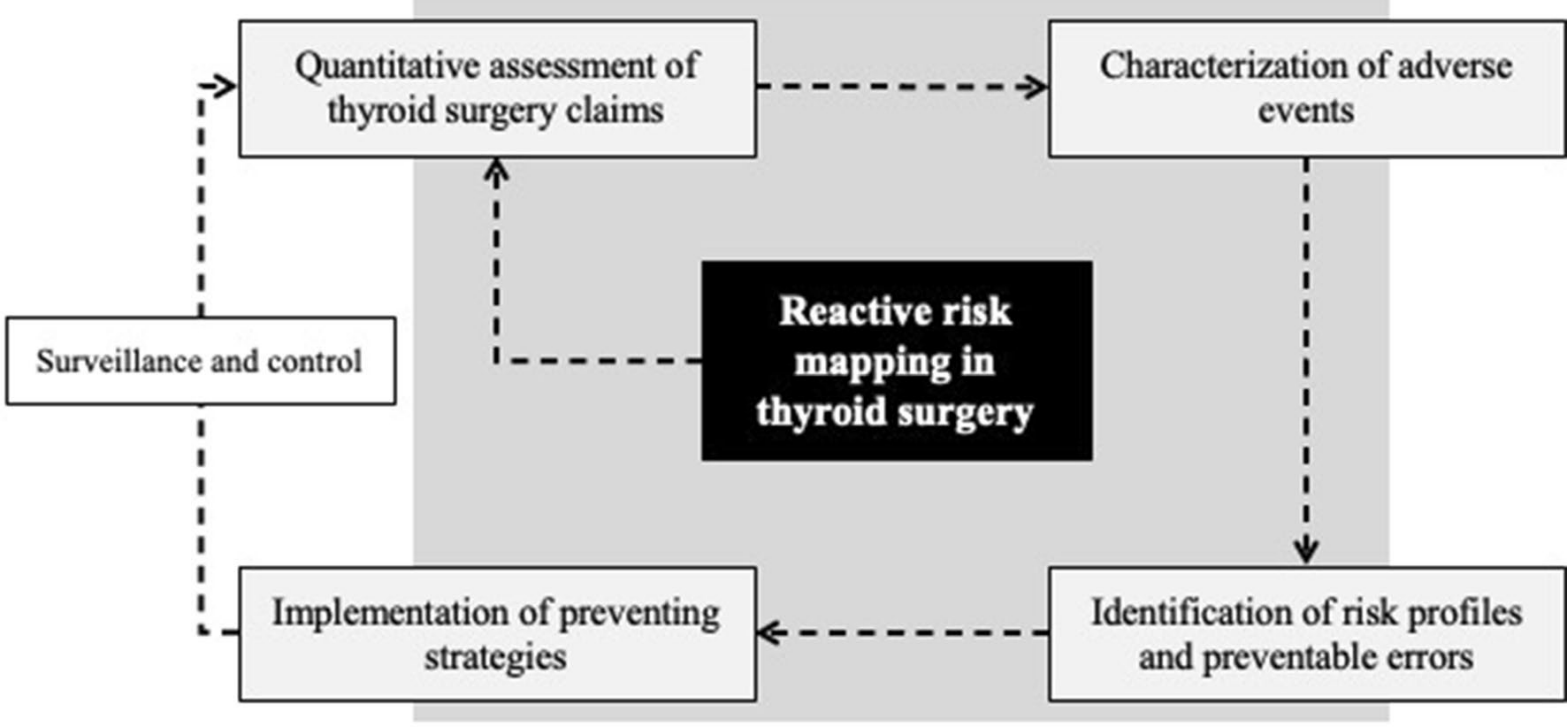

## Introduction

Quality of care assessment is a crucial tool for patient safety implementation. Error represents a critical, intrinsic, and constitutional element with which a health organization is constantly called upon to interface in the context of clinical governance; the risk, with its implications in terms of professional liability and compensation for damage, constitutes the interruption of an ideal path in which all the elements that make up the system are called to integrate and coordinate, and represents the critical point in the determinism of adverse event.

Clinical risk management must be aimed at both the prevention of avoidable errors and the minimization of harmful effects in cases where the risk cannot be eliminated [1]. Risk reduction in an organization translates into a decrease in the incidence of adverse events and sentinel events, as well as a decrease in claims and economic exposure.

Litigation relating to surgical disciplines represents the largest part of the exposure of a healthcare facility in terms of management and economic commitment [2–4]. Thyroid surgery is one of the most numerically represented sectors also due to the continuous increase in interventions. From 2016, more than 40,000 thyroidectomies have been carried out each year in Italy, 80% of which involve the female gender [5]. Between 2004 and 2012, 72,594 patients underwent elective thyroidectomy in England [6]. From data extracted from the Hospital Episodes Statistics (HES) database for England, over the 6-year period (April 2012 to March 2018 inclusive) for all adult thyroidectomy admissions, there are 22,823 total thyroidectomies performed across 144 hospital trusts [7].

Currently, thyroidectomy is indicated as a treatment of choice for benign and malignant thyroid neoplasms, conditions of thyrotoxicosis (in subjects suffering from Graves' disease or other pathologies causing hyperthyroidism) not adequately controlled by medical therapy, resolution of conditions of anatomic-functional alteration secondary to tracheal and/or esophageal compression phenomena, mainly caused by voluminous goiters with retrosternal development (so-called “immersed goiter”); moreover, it is a diagnostic and therapeutic option in case of repeatedly non-diagnostic, indeterminate or suspicious for cancer result at fine needle aspiration cytology (FNAC) [8].

Several studies have shown that most of the disputed events are attributable to incorrect treatment, with a preponderance of recurrent nerve injuries, postsurgical hypoparathyroidism, and information defects [9–13].

Concerning the impact of adherence to the recommendations in reducing the risk of adverse events and litigation, some studies have highlighted the usefulness of the preoperative laryngoscopy study, of exhaustive information about possible complications, as well as of the completeness of the surgical report; on the contrary, the role of intraoperative electrophysiological monitoring of the recurrent nerve as an evaluation parameter of the surgical conduct is not supported by conclusive scientific evidence [14, 15].

In such a context, the experience derived from the management of health liability litigation can represent an important resource in the field of clinical governance, as it can provide data, information, and results of crucial importance in planning care pathways.

Given the significance and topicality of the problem, the present study aims to outline an operational methodology for risk mapping and litigation management in thyroid surgery. Furthermore, the proposed operational approach aims to demonstrate the importance of systematic litigation management as a reactive tool for dealing with clinical risk in a sustainable manner [16–18].

## Methods

The study was conducted through the analysis of data on the activity carried out by the Claims Assessment Committee of Umberto I General Hospital in Rome from 2007 to 2018.

All claims related to thyroid surgery reported during the study period were included and classified based on:

- Categories codified in the International Classification for Patient Safety (ICPS) system [19], suitably modified in relation to the features of the reality under examination; in particular, it was implemented a categorization based on sex and age, operating unit involved, type of event, patient outcome, organizational outcome, presence of internal protocols;
- Clinical features such as underlying thyroid disease, surgical indication, preoperative diagnostic process, informed consent, surgery performed, intraoperative examinations, and postoperative pathway;
- Economic characteristics as requested amount, technical estimate of the claim, risk of loss, and paid amount;
- Chronological references of the main management phases, extrajudicial and judicial.

Subsequently, a descriptive statistical analysis of the categorical variables has been performed with the representation of the frequencies in absolute terms and as a percentage.

## Results

The study involved the analysis of 48 thyroid claims relating to operations performed between 1998 and 2016.

At present, 33 cases (68%) are still open, 8 (17%) have been liquidated following an out-of-court settlement, and 7 (15%) were rejected. Regarding the settled claims, the total economic impact for the hospital amounted to € 261,883, with an average outlay of € 32,735 per claim. The ongoing settlement of many civil proceedings is also linked to the length of trials in Italy. According to the Italian Ministry of Justice, in 2019, the disposition time of civil trials is 379 days and in the second level of judgement, it is 627 days [20].

**Fig. 1:**
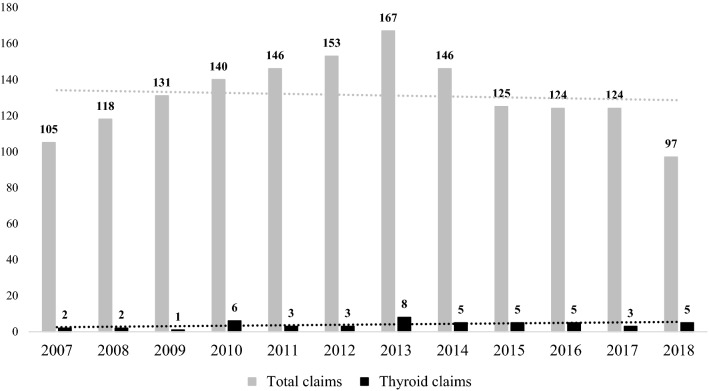
Total claims and thyroid claims frequency during the study period

A preliminary assessment in absolute terms has shown an increasing trend in the number of claims during the study period (Fig. 1). The analysis based on the operating units involved showed a significant dispersion between the departments of general surgery (42; 88%), cancer surgery (1; 2%), thoracic surgery (2; 4%) and ENT surgery (3; 6%). Regarding the type of event, the claims were traced back to the categories of incorrect treatment (44; 92%), blood and blood products (2; 4%) and organizational problems (2; 4%).

Adverse events identified in the inadequate treatment category were unilateral or bilateral recurrent nerve injury (31; 70%); lack of surgical radicality (6; 14%), postsurgical permanent hypoparathyroidism (4; 9%), aesthetic damage due to surgical scars (2; 5%), and dental avulsion during intubation maneuvers (1; 2%).

**Fig. 2:**
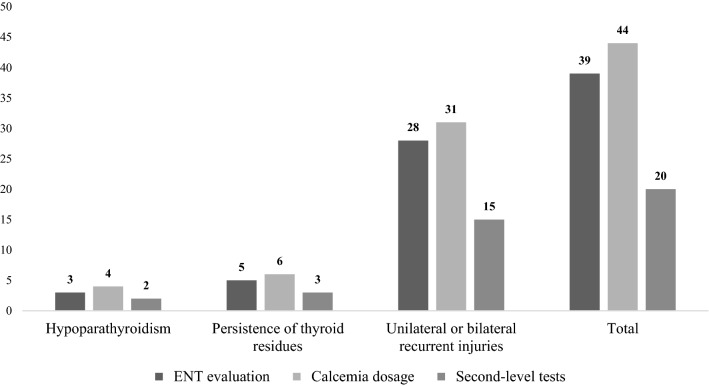
Preoperative tests carried out in the thyroid claims analyzed

The evaluation of claims in relation to compliance with the surgical indications showed substantial coverage of the recommendations in 32 of the 44 cases classified as inadequate treatment. Precisely, it was possible to demonstrate compliance with the indication in only 69% of cases with benign disease while, in the case of malignant thyroid pathology, compliance with the indications for surgery was 78%. Concerning preoperative examinations (Fig. 2), laryngoscopy was performed in 39 (89%) of the 44 claims from inadequate treatment; in the remaining 5 cases, all prior to 2013, it was not possible to document the carrying out of the assessment. The in-depth study limited to the cases of unilateral or bilateral postoperative chordal palsy revealed that this deficiency concerned 3 (9.7%) of the 31 total cases. Regarding the preoperative performance of second-level CT imaging tests, in 5 cases (56%) tests were performed for suspected extrathyroidal involvement, while in the remaining 4 cases (44%) no further instrumental evaluation was performed; in 7 (64%) of 11 cases of voluminous goiter, at least one second-level imaging exam was performed while in the remaining 4 (36%) the surgical intervention planning was based on the indications provided by first-level ultrasound diagnostics.

The quality of the information provided to the patient was assessed by verifying the presence and adequacy of the consent forms for treatment, in relation to compliance with the minimum standards required such as the description of the underlying pathology, type of intervention proposed, complications, therapeutic and alternative options. In most cases (23; 52%) the information proved to be adequate and complete, while in a significant number of cases (21; 48%) criticalities were observed in the information phase; specifically, in 9 cases the consent form was not signed by the physician, in 10 cases the description of the treatment and complications was incomplete or absent, and in 2 cases the informed consent form was even absent.

The analysis of the cases under examination revealed that the quality of the surgical report was in line with the reference standard in 57% of cases. In cases where the operative report was inadequate (19; 43%), the deficiency found consisted in 10 cases in the inadequate description of the times of visualization, isolation, or preservation of the recurrent nerves, while in the remaining 9 cases the surgical report was lacking an adequate description of the technical criticalities found intraoperatively.

Intraoperative electrophysiological monitoring was adopted in 8 (18%) of inadequate treatment claims; while it was not carried out in 36 (82%); of these, 27 claims were attributable to recurrent nerve injuries.

**Fig. 3:**
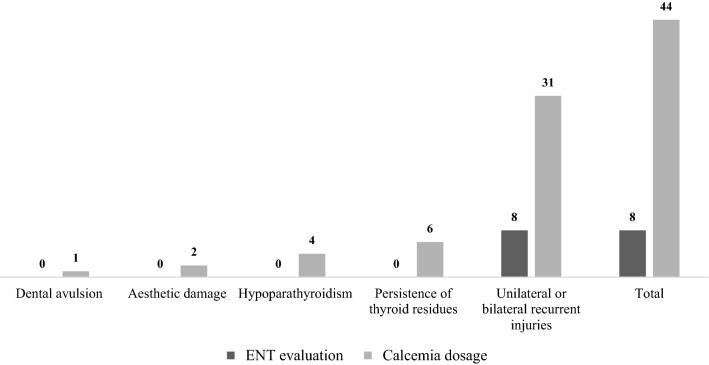
Postoperative tests carried out in the thyroid claims evaluated

Concerning the postoperative path, the evaluation of the chordal function and the respiratory space through laryngoscopy was performed in 26% of claims for recurrent nerve injuries and recommended for discharge in 19% of cases (Fig. 3); in 6 of the 23 cases of chordal paralysis due to iatrogenic lesion of the recurrent nerve, laryngoscopy was indicated during hospitalization, while in 17 cases the examinations were performed independently by the patient and not at postoperative follow-up. The results obtained about the postoperative monitoring of calcium have demonstrated full coverage of the parameter since in all cases there is at least one postoperative calcium dosage, usually performed on the first postoperative day.

**Fig. 4:**
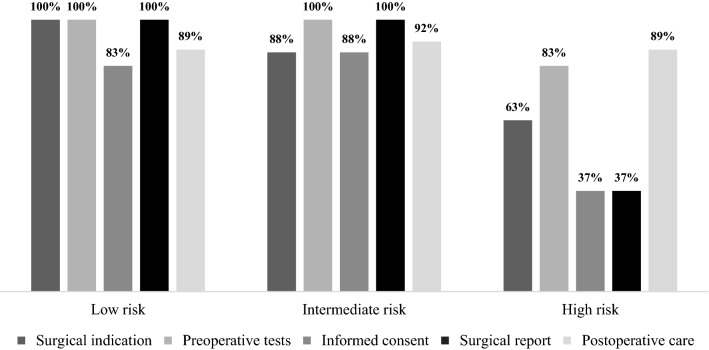
Satisfaction of the indicators in the different categories of risk of loss

Coverage of surgical indications was 63% in high-risk claims, 83% of intermediate-risk claims, and 100% of low-risk claims. The adequacy of the informed consent showed coverage of 37% in high-risk claims, compared to 88% and 83% in moderate and low-risk claims, respectively. Similarly, the surgical report was found to be congruous in 37% of high-risk claims, compared with total adequacy (100%) in moderate and low-risk claims (Fig. 4).

## Discussion

The distribution of complications in thyroid claims made it possible to detect substantial consistency with the data reported in the literature and to implement a reactive approach for the mapping of processes at risk.

Most cases of post-surgical dysphonia and hypoparathyroidism have been observed in case of total thyroidectomy for voluminous goiter, Graves' disease, redo surgery and neoplastic pathology [21].

Within the framework outlined, the operating methodology applied to the analysis of complaints made it possible to identify critical parameters, within which the satisfaction of adequacy criteria has shown absolute importance in the management of disputes and the promotion of preventive measures.

The analysis of the claims based on compliance with the recommended surgical indications showed substantial adherence with requirements. The fulfillment of this parameter has proved to be a fundamental determinant of the risk of loss. The stratification of the data in relation to the diagnosis of acceptance allowed to ascertain how the defects of surgical indication were more frequent in the case of benign pathology. In this study, the stratification of the results obtained in relation to the risk of loss made it possible to ascertain how compliance with the indications, the adequacy of the operating report, and the completeness of the information are extremely important predictive factors. In fact, the satisfaction percentages of these parameters were significantly lower in high-risk claims than in those with moderate or low risk.

Our results reinforce the indication to treat these patients in Thyroid Unit with dedicated surgeons for a correct surgical approach. Based on the data obtained, the strategic need to define internal protocols and operating procedures for the diagnosis and surgical treatment of the different thyroid pathologies is evident. The preoperative otolaryngological evaluation with an examination of the motility of the vocal cords represents a fundamental step in the management of the patient candidate for thyroid surgery; therefore, the preoperative documentation of chordal motility was extremely useful in correctly identifying the real postoperative incidence as well as in the defense in case of litigation [22]. Regarding the preoperative calcium dosage, the examination was routinely performed in all the claims examined and proved to be a useful parameter for assessing the quality of the preoperative process; in the context of the prevention and management of post-surgical hypoparathyroidism, the dosage of the parathyroid hormone could be a further useful examination for the implementation of the preoperative path since it would allow the early identification of patients at greater risk of developing the complication [23]. Similarly, the use of second level imaging methods is a reliable indicator of the quality of preoperative diagnostics; the documentation of the performance of second-level imaging exams for the preoperative characterization of thyroid pathology constitutes an important tool for the planning of diagnostic procedures, the reduction of complications, and the defense in case of litigation [24–26]; the chronological stratification of the data allowed to demonstrate how the progressive preparation of dedicated paths was effective in implementing the quality of preoperative diagnostics despite an inevitable risk margin.

About informed consent, the study highlighted a substantial operational heterogeneity between the units involved in thyroid surgery. The adequacy of the information constitutes a fundamental element both for the defense in case of litigation, but above all for the protection of the right to self-determination and the improvement of patient safety. Therefore, it is desirable to promote measures aimed at standardization through the implementation of a single consent form, elaborated based on the models proposed by scientific societies and adequately integrated based on the experience on the incidence of the most frequent complications.

The quality of the surgical report has proved to be an essential tool for risk mapping in thyroid surgery. The evaluation of the parameter was carried out with respect to the standards proposed by the main surgical scientific societies including the adequacy of the macroscopic description of the thyroid, the reference to the times of direct visualization, isolation and preservation of the recurrent nerves and parathyroid glands, the possible description of macroscopically evident lymphadenopathies or the impossibility to radicalize thyroidectomy, the description of any conditions of increased technical difficulty, and the description of hemostatic procedures. The absent or incomplete description of visualization, isolation, and preservation of the recurrent nerves has generally constituted a suitable element to configure a high risk of loss despite the substantial adequacy of the care path. The results obtained demonstrated the need to adopt hospital policies aimed at pursuing and obtaining greater awareness of the operators concerning the accuracy of the description of the surgical act, especially about the critical moments of the thyroidectomy, such as those represented by direct visualization and preservation of the recurrent nerve.

Consistent with the available evidence, the data obtained show that electrophysiological monitoring of the recurrent nerve is not yet a routine practice due to the recent acquisition and indication in a limited number of cases. In particular, the association of electrophysiological monitoring with direct visualization of nerve structures in cases at higher risk (reoperations, oncological surgery, and approach to voluminous goiters) represents the gold standard for the prevention of iatrogenic lesions of the recurrent nerve [27, 28].

Regarding the postoperative monitoring of chordal function, the present study has shown that there is no codified operative procedure in any of the operative units involved. Laryngoscopy evaluations before discharge were performed only in the presence of dysphonia, aphonia, and dyspnea [29, 30]. The short duration of the postoperative hospital stay was identified as a determinant of the failure to perform the tests. In relation to post-surgical hypoparathyroidism, the study highlighted a substantial operational uniformity between the units with the routine post-operative calcium dosage; this examination allowed the timely recognition of cases of hypocalcemia and the rapid establishment of replacement therapy.

However, the absence of thyroid units and the distribution of interventions between the operating units of general surgery and otolaryngology determine an objective difficulty in standardizing pathways and managing risk.

Conclusively, it is desirable to implement strategic decisions by healthcare facilities aimed at meeting adequacy standards for reducing the risk of adverse events and improving the safety of surgical procedures. Among the quality indicators of healthcare, it is essential to consider the presence of competent operators in thyroid surgery and with documented scientific production, an adequate minimum volume of operational activity, an incidence of postoperative complications in line with the data of international literature. In the context of the virtuous perspective outlined, the promotion of measures aimed at improving the identification of welfare pitfalls through the analysis of litigation has shown effective usefulness in risk management in thyroid surgery. The experience deriving from the retrospective analysis of the litigation from thyroid surgery has made it possible to proceed with accurate mapping of the risks as well as identification of internal standards useful for comparison with the available evidence. Failure to comply with some of these adequacy criteria on the type of complication complained of and on the risk of loss attributed to the accident provided important indications about the direction towards which hospital policies should be directed to improve the safety of treatments in thyroid surgery [31, 32].
